# TGF-**β** drives differentiation of intraepithelial mast cells in inflamed airway mucosa

**DOI:** 10.1172/JCI186337

**Published:** 2025-01-02

**Authors:** Axel Roers

**Affiliations:** Institute for Immunology, University Hospital Heidelberg, Heidelberg, Germany.

## Abstract

Similarly to acute intestinal helminth infection, several conditions of chronic eosinophilic type 2 inflammation of mucosal surfaces, including asthma and eosinophilic esophagitis, feature robust expansions of intraepithelial mast cells (MCs). Also the hyperplastic mucosa of nasal polyposis in the context of chronic rhinosinusitis, with or without COX1 inhibitor intolerance, contains impressive numbers of intraepithelial MCs. In this issue of the *JCI*, Derakhshan et al. elucidate the heterogeneity of MCs in nasal polyposis and identify a transcriptional signature of TGF-β target genes expressed by the intraepithelial MC population. These MCs displayed effector functions that implicate them as pathogenetic contributors. TGF-β directed differentiation of similar MC populations also in vitro. These findings extend the emerging concept of TGF-β as a driver of type 2 inflammation at barrier surfaces.

## Intraepithelial mast cells are elicited by inflammation

**Figure 1:**
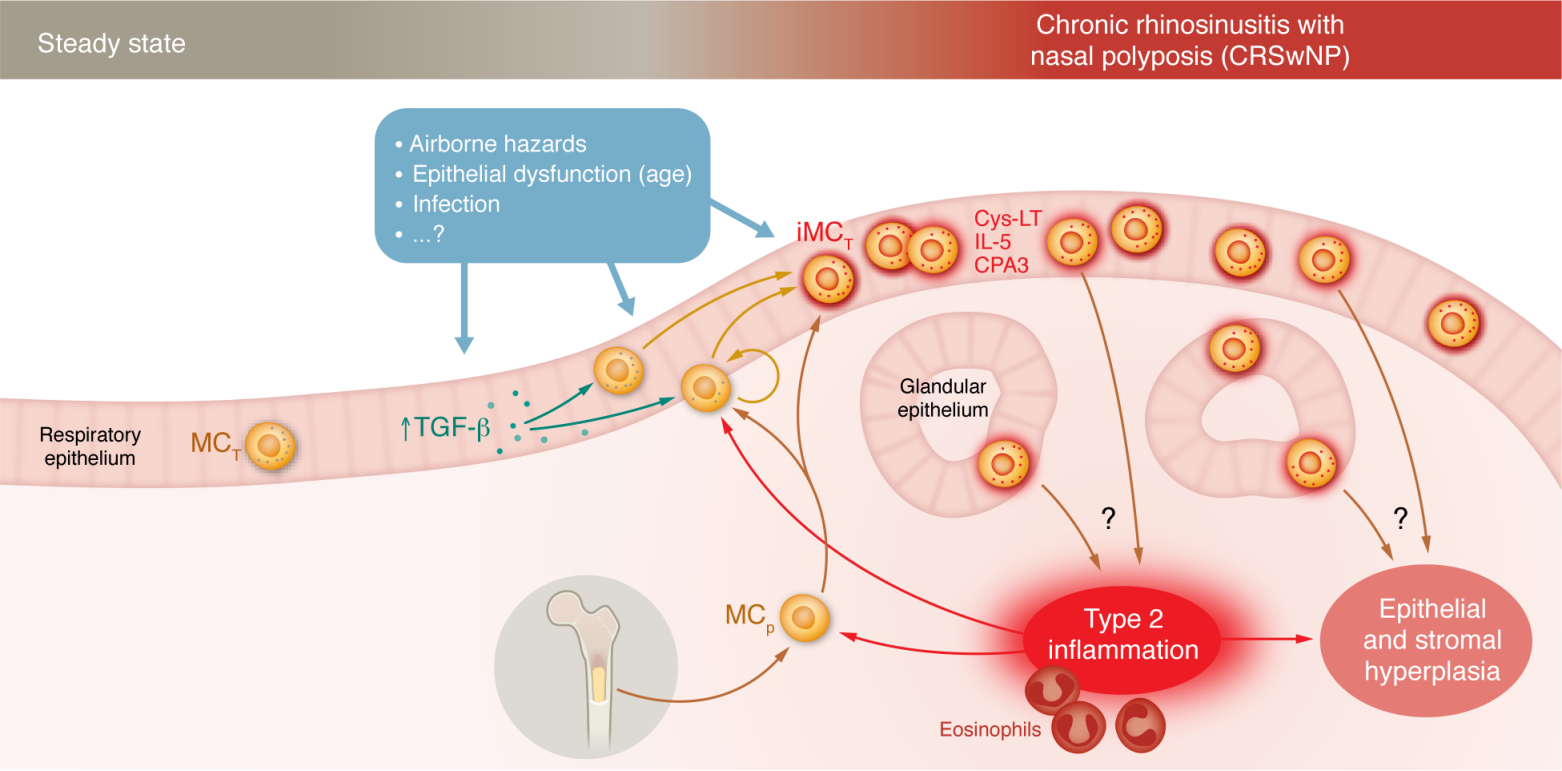
TGF-β directs differentiation of inflammatory intraepithelial MCs in NP. Steady-state nasal mucosa contains few MC_T_ within the respiratory epithelium or in glandular epithelial structures. In CRS, chronic exposure to pathogenetic factors compromises epithelial homeostasis and triggers subepithelial eosinophilic type 2 inflammation. Inflammatory stimuli drive expansion of intraepithelial MC_T_s into a large population by proliferation of MMCs and recruitment of bone marrow–derived MC progenitors (MC_p_). The findings of Derekhshan et al. (1) suggest that stress or damage results in enhanced expression of TGF-β by epithelial cells. TGF-β drives differentiation of an inflammatory MC_T_ phenotype (iMC_T_) with expression of CPA3, IL-5, and increased production of cysteinyl leukotrienes (Cys-LT). MC_T_-derived factors may be key for initiating and/or sustaining the pathogenic type 2 inflammation and hyperplasia of epithelial and stromal compartments.

Mast cells (MCs) are evolutionarily old immune cells, of which humans have 2 largely different types. One is a constitutive component of nonepithelial compartments, e.g., the dermis of the skin or submucosal tissues. These cells originate from fetal progenitors and are self maintained in adult steady-state tissue. They are called MC_TC_s because their secretory granules contain tryptase and chymase (along with cathepsin G and carboxypeptidase). They associate with connective tissue structures, nerves, and blood vessels. Tissue inflammation can result in some increase of MC_TC_ densities. The second population, MC_T_, expressing tryptase but no chymase, are cells of smaller size, primarily located within barrier epithelia. These cells have received less attention for two reasons: (a) they fail to stain reliably by some metachromatic dyes (which readily identify MC_TC_s) and (b) they are sparse in steady-state epithelia. However, they rapidly expand into large populations under the influence of inflammation. In this issue of the *JCI*, Derakhshan et al. (1) report on TGF-β–driven differentiation of inflammatory MC_T_s, which are likely pathogenic in chronic airway disease (Figure 1).

Much of our knowledge about human MC_T_s has been inferred from investigation of their murine equivalent, mucosal MCs (MMCs). The dependence of their presence on inflammatory stimulation is underpinned by their absence in T cell–deficient mice and humans and in germ-free mice. Type 2 inflammation, as in intestinal helminth infection or allergic airway disease, triggers rapid and dramatic IL-4–dependent increases in MMC numbers. While recruitment of hematopoietic stem cell–derived progenitors was considered the major mechanism of this expansion (2), local proliferation also contributes substantially (3). When inflammation ceases and the barrier tissue returns to homeostasis, MMC numbers slowly decline, which may be due to a differentiation program with ultimate downregulation of the growth factor tyrosine kinase receptor KIT that reduces stem cell factor survival signals (2).

## MCs in chronic eosinophilic airway inflammation

While physiological adaptive immune functions of MCs are much debated (4, 5), MCs undisputedly are key pathogenic effector cells of allergic, IgE-driven diseases. Disentangling differential pathogenetic contributions of MC_TC_s versus MC_T_s in IgE-driven disease has been difficult. Combined use of mouse models selectively lacking either connective tissue MCs but not MMCs or lacking all MCs seems an option for teasing out MC subtype functions. Derekhshan et al. (1) investigated human MCs in nasal polyps (NPs) of patients with aspirin-exacerbated respiratory disease (AERD). This aggressive form of chronic airway inflammation features asthma, chronic rhinosinusitis (CRS), and NPs and is associated with an imbalance of mucosal lipid mediator production, which upon iatrogenic COX1 inhibition causes dominance of inflammatory leukotrienes over antiinflammatory prostaglandin E2 and acute airway responses (6). Such NPs have high economic impact often requiring surgical treatment, with high rates of recurrence thereafter. They are associated with atopy and allergic sensitization only in a fraction of patients. The hyperplastic growth of the nasal mucosa is in most cases driven by type 2 chronic eosinophilic inflammation caused by a complex interplay of environmental and microbial factors, age-related changes, and epithelial damage. Normal nasal mucosa and CRS without NPs harbor few MC_T_s, while NPs in the context of CRS, and in particular the NPs associated with AERD, feature dramatically increased numbers of MC_T_s in the polyp epithelia. These cells express CPA3 in addition to tryptase, which seems to reflect a specialized inflammatory MC_T_ program also seen in other conditions of type 2 airway inflammation. MC_TC_s populate stromal compartments of NP tissue in considerable numbers.

## TGF-β–driven MC differentiation

Derakhshan et al. (1) resolved the heterogeneity of MCs in AERD-associated NP tissue and elucidated mechanisms directing differentiation of the MC_T_ population. The group had earlier shown that murine steady-state lung connective tissue MCs and a minor population of MMCs are distinguished by low versus high β7 integrin expression, respectively (7). In those experiments, β7^hi^ MMCs expanded massively with inflammation (7). Correspondingly, the MMC transcriptome included many TGF-β target genes and TGF-β drove differentiation of MMC-like cells in vitro (7). Deep characterization of human MCs from CRS patient-derived nasal polyps (3) revealed a similar transcriptional program in a large NP MC_T_ population that was also expressed in asthma. AERD-associated NPs possessed a proliferating population of cells expressing high levels of CD38 and KIT (CD38^hi^KIT^hi^); they also expressed MC_T_- as well as MC_TC_-signature genes (3). Derakhshan et al. (1) showed that MC_T_s in AERD NP tissue displayed a transcriptional signature of SMAD signaling and TGF-β target genes. TGF-β expression was enhanced in NPs and TGF-β applied in vitro promoted differentiation of MCs toward a MC_T_ phenotype. This program suppressed MC_TC_ signatures but upregulated cytokines, including IL-5, and chemokines as well as enzymes of lipid mediator synthesis, suggesting active pathogenetic contribution. Notably, MC_T_-like cells that were elicited in vitro by TGF-β stimulation and MC_T_s acquired by flow cytometry from human NP tissue readily produced proinflammatory cysteinyl leukotrienes upon IgE crosslinking.

## Evidence of pathogenetic MC contributions

A central question is now whether the dramatically expanded MC_T_ population contributes to NP pathogenesis and drives local type 2 inflammation and hyperplastic mucosal growth. The mere presence of MCs, even in high numbers, cannot be equated with pathogenic function. The impressive MC accumulation in benign human papillomavirus-driven epidermal neoplasia, for example, doesn’t seem to affect epithelial growth (8). In the case of NPs, however, a pathogenetic function of MCs is supported by arguments beyond their numbers and correlation with disease severity. NPs on the background of allergic, but also nonallergic, CRS and NPs in the context of AERD are effectively treated by the anti-IgE antibody omalizumab, which reduces activation of MCs (9). MCs are a major IgE-responsive cell type in NP tissue, albeit not the only one, as also activated eosinophils, for example, express FcεRI and can be stimulated via surface IgE. In AERD-associated polyposis, anti-IgE therapy also reduces lipid mediator release upon challenge with nonsteroidal antiinflammatory drugs (10). NP MC_T_s are an important source of lipid mediators as shown by Derakhshan et al. (1) Furthermore, anti–IL-5 treatment is beneficial in NP, and MC_T_s were identified as the major source of IL-5 in NP tissue. A recent report describes MC_T_s triggering type 2 immunity at mouse intestinal mucosa by direct induction of enterocyte IL-33 release (11).

## Implication for therapy of nasal polyposis

Future therapeutic approaches targeting NP MCs may include blockade of KIT by topically administrated tyrosin kinase inhibitors (TKIs), which would avoid adverse effects of systemic KIT inhibition. This strategy may not enable reduction of NP MC numbers, as MC burden in asthma was only marginally reduced by systemic TKI treatment (12). However, recent experiments show that KIT inhibition reduces MC responsiveness and systemic TKI suppresses in vivo anaphylactic reactions in mice (13). New principles might enable entirely new concepts of future therapeutic MC depletion, including inhibitors of carbonic anhydrase 1 (14). The work of Derekhshan et al. (1) now suggests pharmacologic interference with the TGF-β signals driving expansion and differentiation of induced MC_T_ populations in NP tissue as a therapeutic concept. While best known for its immunosuppressive capacity, TGF-β signals are increasingly recognized as essential drivers of type 2 inflammation (15). Inhibitors of TGF-β activation or receptor binding as well as blockers of TGF-β receptor signaling are under development for various conditions, including fibrosis and cancer. Topical administration of small molecules inhibiting TGF-β receptor signaling might prove an effective strategy to suppress the pathogenic type 2 inflammation of NP tissue and at the same time directly block induction of the potential key malefactor MC_T_ population. As TGF-β has complex homeostatic effects on tissues and can on the one hand promote regeneration but on the other suppress neoplastic growth, direct impacts of pharmacologic interference with TGF-β on the epithelial hyperplasia and stromal hyperplasia that constitute NPs are difficult to predict. Animal models of chronic eosinophilic rhinosinusitis and polyposis may provide clues here.

Understanding mechanisms driving MC hyperplasia and directing their differentiation in chronic eosinophilic airway inflammation will instruct the design of therapies targeting these heavily incriminated cells. Therapies eliminating MCs or interfering with their differentiation or function in nasal polyposis will also ultimately clarify the extent to which MCs contribute to the pathogenesis of this difficult-to-treat condition.

## Version 1 01/02/2025

Electronic publication
